# Classification of Five Uremic Solutes according to Their Effects on Renal Tubular Cells

**DOI:** 10.1155/2014/512178

**Published:** 2014-11-09

**Authors:** Takeo Edamatsu, Ayako Fujieda, Atsuko Ezawa, Yoshiharu Itoh

**Affiliations:** Pharmaceutical Division, Kureha Corporation, 3-26-2 Hyakunin-cho, Shinjuku-ku, Tokyo 169-8503, Japan

## Abstract

*Background/Aims*. Uremic solutes, which are known to be retained in patients with chronic kidney disease, are considered to have deleterious effects on disease progression. Among these uremic solutes, indoxyl sulfate (IS) has been extensively studied, while other solutes have been studied less to state. We conducted a comparative study to examine the similarities and differences between IS, *p*-cresyl sulfate (PCS), phenyl sulfate (PhS), hippuric acid (HA), and indoleacetic acid (IAA). *Methods*. We used LLC-PK1 cells to evaluate the effects of these solutes on viable cell number, cell cycle progression, and cell death. *Results*. All the solutes reduced viable cell number after 48-hour incubation. N-Acetyl-L-cysteine inhibited this effect induced by all solutes except HA. At the concentration that reduced the cell number to almost 50% of vehicle control, IAA induced apoptosis but not cell cycle delay, whereas other solutes induced delay in cell cycle progression with marginal impact on apoptosis. Phosphorylation of p53 and Chk1 and expression of ATF4 and CHOP genes were detected in IS-, PCS-, and PhS-treated cells, but not in IAA-treated cells. *Conclusions*. Taken together, the adverse effects of PCS and PhS on renal tubular cells are similar to those of IS, while those of HA and IAA differ.

## 1. Introduction

Uremic solutes are a large number of compounds that are retained in chronic kidney disease (CKD), especially in end-stage renal disease, resulting in elevated serum concentrations compared to normal condition [1]. These solutes are excreted in urine in healthy persons but are accumulated as CKD progresses [2, 3]. Over a hundred uremic solutes have been reported to date, and these solutes are classified into three groups according to the size and protein binding properties [4–6]. Protein-bound solutes have attracted much attention in the last decade, because they are less efficiently removed by dialysis [7] and are possibly associated with CKD-related complications [8].

Indoxyl sulfate (IS) is a representative protein-bound solute and its deleterious effects have been studied in various cell types including renal tubular cells [9], mesangial cells [10], vascular endothelial cells [11], vascular smooth muscle cells [12], osteoclasts [13], osteoblasts [14], erythrocytes [15], and monocytes [16]. Moreover, several studies demonstrate the adverse effects of IS on the kidney [17, 18] and vascular systems [19, 20] of animal models. In humans, several reports indicate the association of IS with impairment of renal function and development of cardiovascular disease in CKD patients [21–23]. Although these studies imply a causal relationship between IS and progression of CKD and/or CKD-related complications, it is necessary to clarify whether other uremic solutes have similar effects.

Our previous study demonstrated that the serum levels of several solutes are elevated in CKD rats [24]. Most of these solutes are also found to be increased in hemodialysis patients [25]. Among these solutes, we focused on five solutes which were IS,* p*-cresyl sulfate, phenyl sulfate, hippuric acid, and indoleacetic acid. The selection criteria for these solutes were both their high serum concentration in hemodialysis patients and their effect on viable cell number of porcine renal tubular cells (unpublished observation).

Renal tubular cells are the cell component of renal tubules, and tubular injury is thought to be one of the key events causing progression of CKD [26]. Thus we evaluated the effects of the five uremic solutes on viable cell number of renal tubular cells and investigated the underlying mechanisms.

## 2. Materials and Methods

### 2.1. Cell Culture

LLC-PK1, a porcine renal tubular epithelial cell line, was obtained from American Type Culture Collection (ATCC, Manassas, VA, USA). LLC-PK1 cells were maintained in Medium 199 (Mediatech, Manassas, VA, USA) supplemented with 10% FBS (BioWest, Nuaillé, France).

### 2.2. Reagents

Indoxyl sulfate was purchased from Biosynth (Staad, Switzerland).* p*-Cresyl sulfate and phenyl sulfate were synthesized at Eiweiss (Shizuoka, Japan). Hippuric acid and indoleacetic acid were purchased from Tokyo chemical industry (Tokyo, Japan). N-Acetyl-L-cysteine was purchased from Sigma (St. Louis, MO, USA).

### 2.3. Viable Cell Count

Effects of uremic solutes on viable cell number were determined using the Cell Counting Kit-8 (Dojindo, Wako, Tokyo, Japan), a water-soluble version of the methyl thiazolyl tetrazolium assay, according to the manufacturer's instructions. LLC-PK1 cells were suspended in Medium 199 supplemented with 2% FBS and dispensed into tubes. Each uremic solute was added to the cell suspension and cell density was adjusted to 1.6 × 10^4^ cells/mL. One milliliter/well of the cell suspension was seeded in a 24-well plate and incubated for 48 hours. After incubation, the medium was changed to a medium containing the Cell Counting Kit-8 reagent. The cells were incubated for another 30 min and the optical density at 450 nm (OD450) was measured using a microplate reader (iMark Microplate Reader, Bio-Rad, Hercules, CA, USA). The OD450 of medium containing Cell Counting Kit-8 reagent without cells was measured and was subtracted from the OD450 of each sample.

### 2.4. Cell Cycle Analysis

Effects of uremic solutes on growth rate were evaluated after adjusting the cell cycle at G1/S or G2/M boundary. Double thymidine block was used for synchronization at G1/S boundary. Cells were incubated overnight in serum-free medium containing 2.5 mmol/L of thymidine, changed to medium supplemented with 10% FBS, and incubated for 7 hours and then in thymidine-containing medium again overnight. For G2/M synchronization, cells were incubated overnight in serum-free medium containing 1.0 *μ*mol/L of nocodazole. After synchronization, the cells were treated with uremic solutes as described above for 4 or 6 hours. Then, the cells were fixed in ethanol and stained with propidium iodide (Propidium Iodide/RNase Staining Solution, Cell Signaling Technology, Danvers, MA, USA). The stained cells were detected by flow cytometer (FACS Calibur, Becton Dickinson, Franklin Lakes, NJ, USA) and the data were analyzed using the Flowjo software (Tomy Digital Biology, Tokyo, Japan).

### 2.5. Apoptosis

Cells were treated with uremic solutes as described above for 48 hours. After treatment, culture medium that might contain nonadherent cells and adherent cells was harvested. The cells were stained with FITC-conjugated annexin V and propidium (TACS Annexin V-FITC Apoptosis Detection Kit, Trevigen, Gaithersburg, MD, USA). The stained cells were detected by flow cytometer (FACS Calibur) and the data were analyzed using the Flowjo software.

### 2.6. Western Blotting

Cells were treated with uremic solutes as described above for 5 hours. After treatment, the cells were lysed with RIPA Lysis Buffer (Santa Cruz Biotechnology, Santa Cruz, CA, USA). Antibodies against phosphorylated p53 (at Ser15) and Chk1 (at Ser345) were used (both are supposed to be active forms and were purchased from Cell Signaling Technology). Chemiluminescent signals generated using the ECL Select Western Blotting Detection System (GE Healthcare, Buckinghamshire, UK) were detected by Light-Capture II Cooled CCD Camera Systems (ATTO, Tokyo, Japan). Antibody against *β*-actin was used as loading control (Biolegend, San Diego, CA, USA).

### 2.7. Real-Time PCR

Cells were treated with uremic solutes as described above for 5 or 24 hours. After treatment, the cells were lysed in ISOGEN (Nippon Gene, Tokyo, Japan). Total RNA was isolated according to the manufacturer's instructions, and optical density at 260 nm was measured using a spectrophotometer (Gene Spec V, Hitachi High-Tech Manufacturing & Service Corporation, Ibaraki, Japan). The RNA (300 ng) was reverse-transcribed using the PrimeScript RT Reagent Kit (Takarabio, Shiga, Japan) in a 10 *μ*L reaction volume. Using 0.6 *μ*L of complementary DNA, SYBR Premix Ex Taq II Tli RNaseH Plus (Takarabio), and 10 pmol of each of the primer sets for various target genes (Table 1), real-time PCR was performed in a 25 *μ*L reaction volume and analyzed using the Thermal Cycler Dice Real Time System (TP800, Takarabio) at the following thermal cycling conditions: denaturation at 95°C for 30 sec, followed by 40 cycles of 95°C denaturation for 5 sec and 60°C annealing/extension for 30 sec.

### 2.8. Statistical Analysis

Statistical analysis was performed by Student's *t*-test, and *P* < 0.01 was considered as significant.

## 3. Results

### 3.1. Effects on Cell Viability

**Figure 1:**
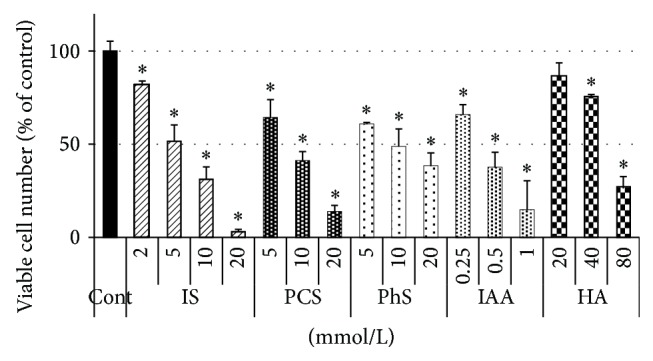
Effects of five uremic solutes on viable cell number. Porcine renal tubular cells were treated with each uremic solute for 48 hours. Viable cell number was evaluated using Cell Counting Kit-8. Optical density at 450 nm was measured and the values were calculated as percent of control. Data are shown as mean ± S.D (*n* = 4). Asterisks indicate significant reduction of viable cell population compared to control (*P* < 0.01). Cont, control; IS, indoxyl sulfate; PCS,* p*-cresyl sulfate; PhS, phenyl sulfate; HA, hippuric acid.

All five uremic solutes decreased viable cell number dose-dependently upon incubating with LLC-PK1 cells for 48 hours (Figure 1). To compare the mechanisms of action of the five solutes, the concentration of each solute that reduces the cell number by almost 50% was used in subsequent experiments. For IAA, two or three different concentrations were always used, because the reduction rates differed in different experiments. In some experiments, two concentrations of IS were used for the same reason. Viable cell numbers were evaluated in each experiment for confirmation purpose. The results of viable cell numbers for various experimental conditions are shown in Supplementary Figures available online at http://dx.doi.org/10.1155/2014/512178.

### 3.2. Effects on Cell Cycle Progression

The decrease in viable cell number is supposed to be due to either reduction in growth rate or induction of cell death. To investigate these two possibilities, the effects of uremic solutes on growth rate and apoptosis were evaluated.

**Figure 2:**
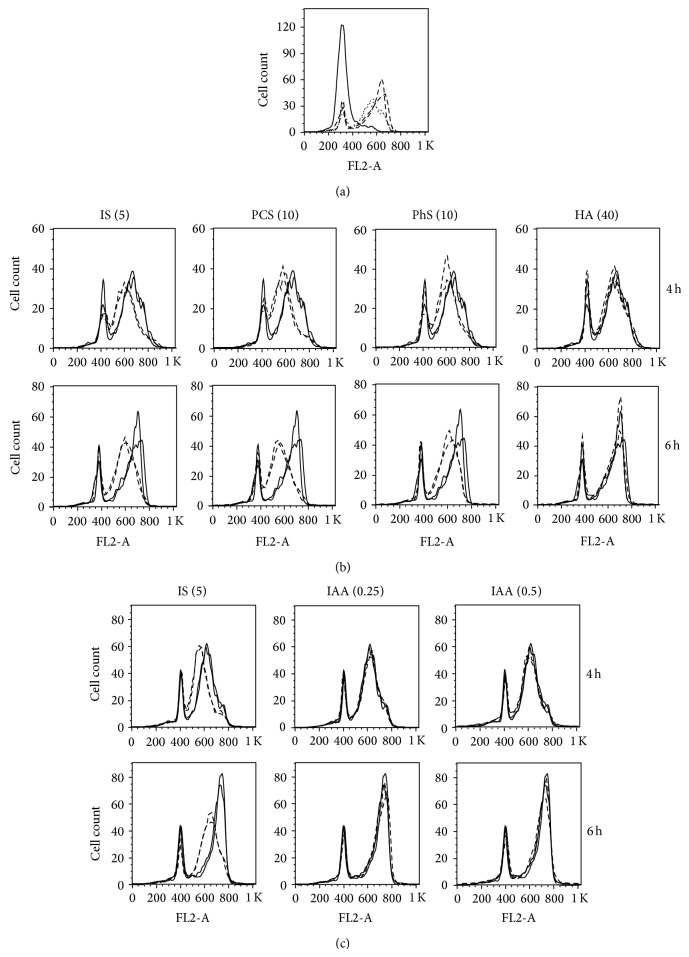
Effects of uremic solutes on cell cycle progression after porcine renal tubular cells were synchronized at G1/S boundary. (a) Time-dependent shift of histograms of untreated control cells. Each line indicates time after release from synchronization. Solid line: 0 hours, dotted line: 4 hours, and dashed line: 6 hours. Duplicate data for each time point are shown except for 0 hours. (b) and (c) Histograms of uremic solute-treated cells and corresponding control cells at indicated time points. After synchronization, cells were released to grow with (dashed line) or without (solid line) uremic solute for indicated periods. Cont, control; IS, indoxyl sulfate; PCS,* p*-cresyl sulfate; PhS, phenyl sulfate; HA, hippuric acid; IAA, indoleacetic acid. Concentrations of solutes (mmol/L) are shown in parentheses. Duplicate data are shown. Data are representative of two independent experiments.

First, to determine the effects on growth rate, cells were synchronized at G1/S boundary, and the synchronized cells were released to grow with or without uremic solutes. In this experimental setting, the cell cytometry histogram shifts from left to right in a time-dependent manner, according to the increase in DNA content (Figure 2(a)). Therefore, delayed growth will appear as a lag of the histogram on the left hand side of the corresponding control. Under these experimental conditions, IS,* p-*cresyl sulfate (PCS), and phenyl sulfate (PhS) delayed growth rate (Figure 2(b)), whereas hippuric acid (HA) and IAA (Figure 2(c)) had no impact on growth rate. In this experiment, 0.25 and 0.5 mmol/L of IAA decreased viable cell number by 26 and 84%, respectively (Supplementary Figure 1).

**Figure 3:**
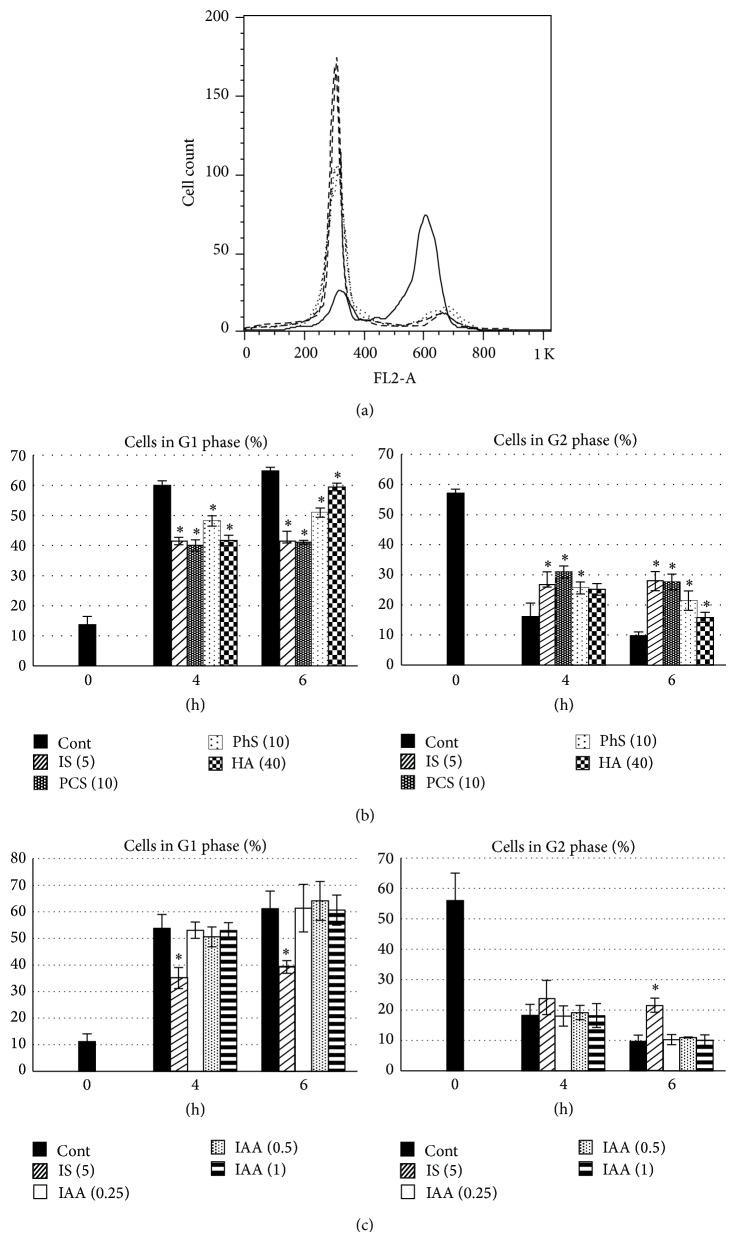
Effects of uremic solutes on cell cycle progression after porcine renal tubular cells were synchronized at G2/M boundary. (a) Time-dependent shift of histograms of untreated control cells. Each line indicates time after release from synchronization. Solid line: 0 hours, dotted line: 4 hours, and dashed line: 6 hours. Duplicate data for each time point are shown except for 0 hours. (b) and (c) Percent of cells in G1 or G2 phase. After synchronization, cells were released to grow with or without uremic solutes for indicated periods. Percent of cells in each phase was analyzed and calculated. Cont, control; IS, indoxyl sulfate; PCS,* p*-cresyl sulfate; PhS, phenyl sulfate; HA, hippuric acid; IAA, indoleacetic acid. Data are shown as mean ± S.D. (*n* = 4). Asterisks indicate significant difference compared to corresponding control (*P* < 0.01). Concentrations of solutes (mmol/L) are shown in parentheses.

Next, cells were synchronized at G2/M boundary, and then the cells were released to grow with or without uremic solutes. In this experimental setting, the histogram of untreated control cells at 0 hours shows a predominant cell population on the right (cells in G2 phase, Figure 3(a)) with negligible amount on the left (cells in G1 phase). As mitosis progresses, cells in G2 phase decrease and cells in G1 phase increase. Data are shown as percent of cells in G1 and G2 phases. IS, PCS, PhS, and HA retarded both the decrease of cells in G2 phase and the increase of cells in G1 phase (Figure 3(b)). In sharp contrast to these four solutes, IAA had no effect on the rate of G2 to G1 transition (Figure 3(c)). In this experiment, 0.25, 0.5, and 1 mmol/L of IAA decreased viable cell number by 12, 42, and 79%, respectively (Supplementary Figure 2).

### 3.3. Effects on Cell Death

**Figure 4:**
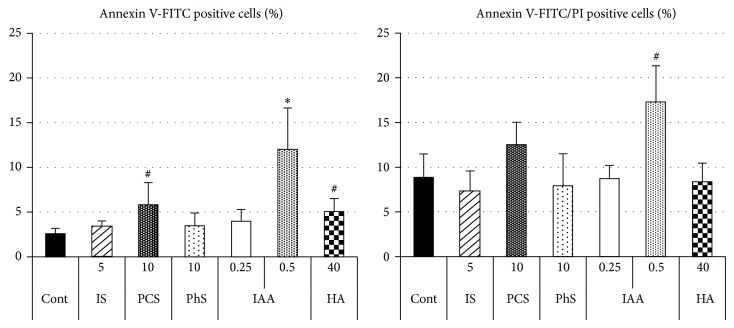
Effects of uremic solutes on cell death. After 48-hour incubation with each uremic solute, porcine renal tubular cells were stained with annexin V-FITC and propidium iodide (PI). Percent of annexin V-positive cells and annexin V/PI-positive cells was analyzed and calculated. Cont, control; IS, indoxyl sulfate; PCS,* p*-cresyl sulfate; PhS, phenyl sulfate; HA, hippuric acid; IAA, indoleacetic acid. Data are shown as mean ± S.D. (*n* = 4). Asterisk indicates significant difference compared to corresponding control (*P* < 0.01). Sharp marks (#) indicate *P* < 0.05.

Because IAA had no effects on growth rate as described above, the effect on cell death was examined. Annexin V-FITC and propidium iodide were used to detect dying and dead cells, respectively. As a result, only IAA induced significant increase of dying cells (Figure 4), while marginal increases of dying cells were observed in PCS or HA-treated groups (*P* = 0.044 and 0.021, resp.). IAA also induced marginal increase of dead cells (*P* = 0.013).

### 3.4. Inhibition of Cell Viability Reduction by Antioxidant

**Figure 5:**
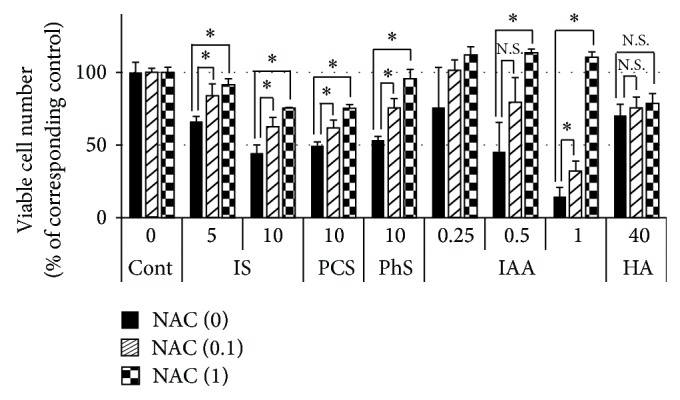
Effect of N-acetyl-L-cysteine (NAC) on uremic solute-induced decrease in viable cell number. Porcine renal tubular cells were treated with NAC (0, 0.1 or 1 mmol/L) for 20 minutes and further treated with each uremic solute for 48 hours. Viable cell number was evaluated using Cell Counting Kit-8. Optical density at 450 nm was measured and the values were calculated as percent of the corresponding control. Cont, control; IS, indoxyl sulfate; PCS,* p*-cresyl sulfate; PhS, phenyl sulfate; HA, hippuric acid; IAA, indoleacetic acid. Data are shown as mean ± S.D. (*n* = 4). Asterisks indicate significant difference each pairs described in this figure (*P* < 0.01). N.S. means nonsignificant difference.

Previous studies have reported that IS induces reactive oxygen species (ROS) and that ROS could be involved in IS-induced inhibition of cell proliferation in several cell types [27, 28]. Therefore, we examined the effect of N-acetyl-L-cysteine, an antioxidant, on reduction of viable cell number induced by each solute. Incubation with N-acetyl-L-cysteine partially inhibited reduction of viable cell number in IS, PCS, or PhS groups and completely inhibited the reduction in IAA group (Figure 5). Meanwhile, N-acetyl-L-cysteine did not inhibit the effect of HA.

### 3.5. Phosphorylation of p53 and Chk1

**Figure 6:**
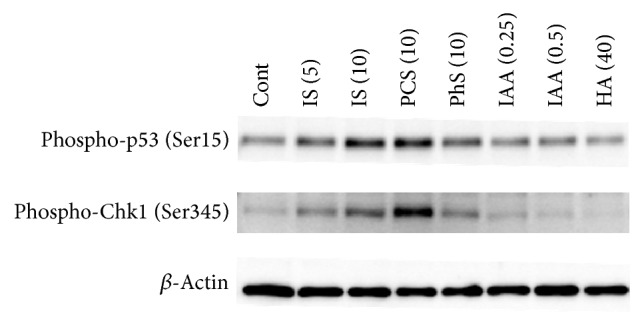
Effects of uremic solutes on p53 and Chk1 phosphorylation. Porcine renal tubular cells were treated with each uremic solute for 5 hours. Cell lysates were obtained and subjected to Western blotting. Cont, control; IS, indoxyl sulfate; PCS,* p*-cresyl sulfate; PhS, phenyl sulfate; HA, hippuric acid; IAA, indoleacetic acid. Images are representative of two independent experiments.

IS has been reported to inhibit cellular proliferation in a p53-dependent manner in HK-2, a human proximal tubular cell line [29]. Therefore, we examined phosphorylation of p53. We also examined phosphorylation of Chk1 that is also involved in DNA damage response like p53. The results showed that IS and PCS induced p53 phosphorylation (Figure 6), while IS, PCS, and PhS induced Chk1 phosphorylation. IAA and HA had no effect on phosphorylation of these proteins. In this experiment, 0.25 and 0.5 mmol/L of IAA decreased viable cell number by 30 and 55%, respectively, and 5 and 10 mmol/L of IS decreased viable cell number by 42 and 76%, respectively (Supplementary Figure 4).

### 3.6. Expression of Genes Involved in Growth Delay or Cell Death

**Figure 7:**
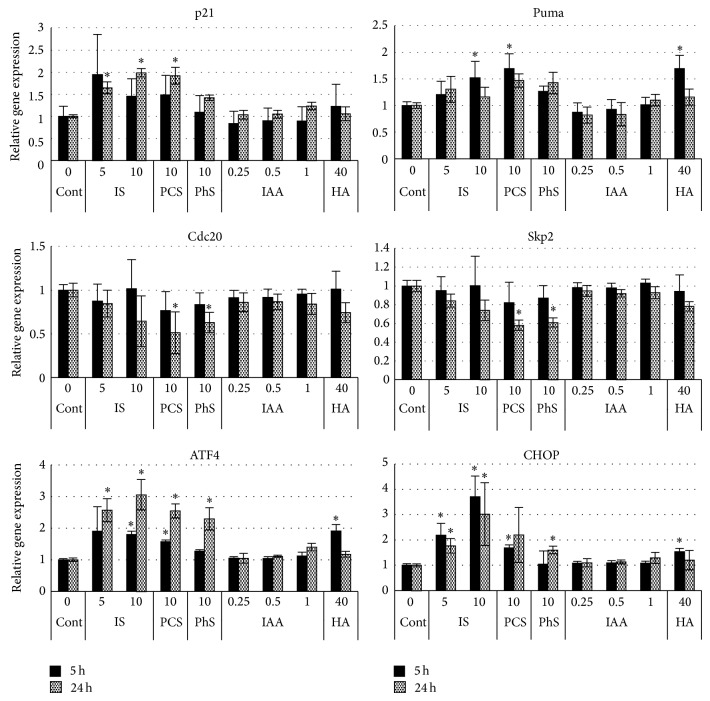
Effects of uremic solutes on gene expression. Porcine renal tubular cells were treated with each uremic solute for 5 and 24 hours. Total RNA was extracted and subjected to real-time PCR. Gene expression level relative to GAPDH expression was calculated by ΔΔCt method. Data are expressed as relative values to the corresponding control. Cont, control; IS, indoxyl sulfate; PCS,* p*-cresyl sulfate; PhS, phenyl sulfate; HA, hippuric acid; IAA, indoleacetic acid. Data are shown as mean ± S.D. (*n* = 6). Asterisks indicate more than 1.5-fold increase or decrease with statistical significance compared to corresponding control (*P* < 0.01).

To further explore molecules which are involved in uremic solute-induced cell cycle delay and/or cell death, we examined the expression of genes known to play important roles in these processes (Figure 7). IS and PCS significantly induced p21 mRNA expression at 24 hours after treatment. IS (10 mmol/L), PCS, and HA significantly induced Puma (p53 upregulated modulator of apoptosis) mRNA expression at 5 hours after treatment. PCS and PhS suppressed Cdc20 and Skp2 mRNA expression at 24 hours after treatment. IS, PCS, PhS, and HA induced ATF4 and CHOP mRNA. On the other hand, IAA did not affect the expression of all these genes at all concentrations tested. In this experiment, 0.25, 0.5, and 1 mmol/L of IAA decreased viable cell number by 16, 49, and 72%, respectively (Supplementary Figure 5).

## 4. Discussion

**Figure 8:**
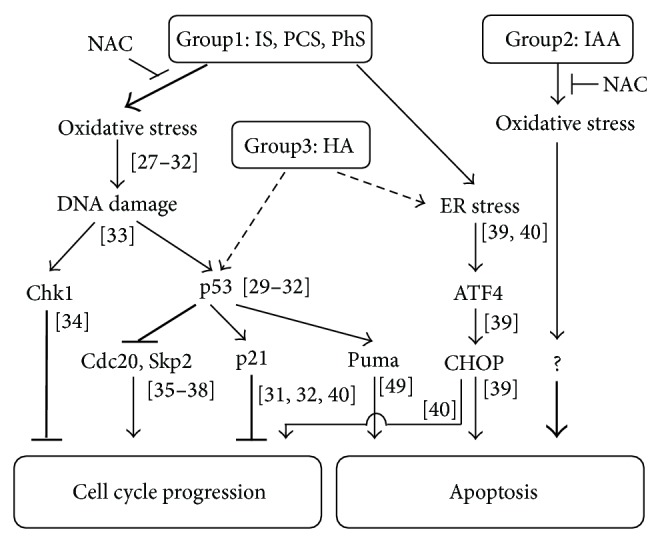
Schematic diagram of the effects of uremic solutes on porcine renal tubular cells.* Group 1* exclusively induces cell cycle delay, which would be mediated by oxidative stress and ER stress. IS is reported to induce oxidative stress [27–32] and ER stress [40]. Oxidative stress can cause DNA damage, which is followed by Chk1 and p53 activation [33]. Activated Chk1 can arrest cell cycle progression [34]. Activation of p53 by IS is also reported [29–32]. Activated p53 can repress transcription of Cdc20 [38] and Skp2 [36, 37]. Because both Cdc20 and Skp2 have important roles in cell cycle progression [35], their repression might delay cell cycle. Another downstream target of p53, p21, which is also an important regulator of cell cycle, is also reported to be induced by IS [31, 32, 40]. Meanwhile, ER stress can induce ATF4 and CHOP expression, which is followed by apoptosis [39]. However, it is also reported that IS induced CHOP expression and CHOP mediates inhibition of proliferation instead of induction of apoptosis in human renal tubular cells [40]. Thus, observed ATF4 and CHOP mRNA induction might be involved in cell cycle delay rather than apoptosis.* Group 2* exclusively induces apoptosis, but its mechanism of action is largely unknown.* Group 3* marginally induces both cell cycle delay and apoptosis, which might be partly mediated by p53 and ER stress. More detailed explanation is in Section 4. Cont, control; IS, indoxyl sulfate; PCS,* p*-cresyl sulfate; PhS, phenyl sulfate; HA, hippuric acid; IAA, indoleacetic acid; NAC, N-acetyl-L-cysteine. Number in brackets corresponds to the number of reference literature.

To the best of our knowledge, this is the first study that systematically compared the effects of five uremic solutes on porcine renal tubular cells. Our results are schematized in Figure 8. The five solutes evaluated in this study were categorized into three groups by the difference in mechanism of reducing viable cell number upon 48-hour treatment.

The first group is composed of IS, PCS, and PhS. This group of solutes induces delay in cell cycle progression rather than cell death to reduce the viable cell number. Oxidative stress is presumably involved in these processes, because viable cell number was partly recovered by incubation with N-acetyl-L-cysteine. Moreover IS is reported to induce oxidative stress in several cell types including renal tubular cells [27–32]. This oxidative stress would induce DNA damage, which is followed by activation of Chk1 and p53 [33]. Subsequent modulation or regulation of their downstream targets would eventually delay cell cycle progression [31, 32, 34–38]. Meanwhile, upregulation of ATF4 and CHOP, known as a hallmark of one of the three pathways of endoplasmic reticulum stress (ER stress) response, was also observed. ER stress response is an adaptive response in nature, but prolonged ER stress overwhelms the adaptive response and eventually results in apoptosis through CHOP upregulation [39]. Thus, upregulation of these genes could induce apoptosis, but only marginal increase of dying cells was observed with this group of solutes. A possible reason is that the incubation time was too short to observe apoptosis. On the other hand, it is reported that CHOP induction by IS inhibits cellular proliferation in human proximal tubular cells [40]. Therefore upregulation of ATF4 and CHOP might also contribute to delay in cell cycle progression in addition to activation of the p53 pathway.

IS has been reported to inhibit proliferation of human proximal tubular cells through inducing oxidative stress, p53 activation, and ER stress [29, 32, 40]. The present study confirms these phenomena in porcine tubular cells and additionally reveals that PCS and PhS induce growth retardation in a similar manner as IS. Administration of PCS to CKD rat model has been shown to cause further renal tubular damage by mechanisms similar to that of IS [41]. Moreover another report reveals that IS and PCS induce similar inflammatory gene expressions in renal tubular cells [42]. Thus, IS, PCS, and PhS might aggravate renal function via p53 activation and ER stress in an additive manner. Activation of p53 and resultant cellular senescence have been proposed to increase sensitivity to insult and decrease repair ability in the renal system, further impairing renal function [43]. ER stress is also known to be involved in the progression of kidney disease [44]. Future studies are necessary to clarify the involvement of these solutes in the induction of cellular senescence and/or ER stress in clinical settings.

IS also induces cellular senescence via p53 activation in other cell types such as endothelial cells [30] and vascular smooth muscle cells [31], which implies involvement of IS in the progression of cardiovascular disease (CVD) in CKD patients, as has been suggested in several epidemiological studies [22, 23]. The association of PCS with CVD has also been reported [45–47]. Thus it would be interesting to evaluate the effects of PCS and PhS in vascular cells.

The second group comprises only IAA, but other solutes not tested in this study may also belong to this group. In sharp contrast to IS, IAA induces cell death rather than delay of cell cycle progression to reduce viable cell number. Although the effects of both IAA and IS seemed to be mediated by ROS, the results were different. This might be due to the difference of subcellular organelle where ROS production takes place. However, future studies are required to clarify the mechanisms in detail. Interestingly, senescent endothelial cells are more susceptible to apoptotic stimuli [48]. Thus one might consider the possibility that IS (a cellular senescence-inducing solute) and IAA (a cell death-inducing solute) may affect viability of certain cell types in a cooperative manner.

The third group comprises HA, but again other solutes may also belong to this group. HA marginally induces delay both in cell cycle progression and in cell death, which differs from the other two groups. N-Acetyl-L-cysteine did not inhibit the effect of HA, further supporting the notion that HA forms another group. Although phosphorylation of p53 was not observed in HA-treated cells, HA did modulate the expression of p53-regulated genes (Puma) [49] to some extent. Thus HA may induce p53 activation slightly. Furthermore, HA would induce ER stress, as indicated by upregulation of the marker gene ATF4 and CHOP. These results suggest that HA may affect the cellular system in a somewhat overlapping manner with the first group.

This study has some limitations. First, the concentrations of the uremic solute tested in this experiment were much higher than the serum concentrations in CKD patients [2, 5, 25]. In clinical settings, multiple uremic solutes exist and may affect biological systems in an additive or synergistic manner. However, only a single solute was tested in each experiment. Thus, high concentrations were necessary to obtain measurable effects on cellular function and to evaluate the mechanisms of each solute.

In conclusion, this study suggests that uremic solutes can be categorized into groups depending on their mechanisms of action on cells. We speculate that solutes within a group exert their effects in an additive manner while solutes in different groups act in a cooperative manner. Future studies are necessary to verify these possibilities.

## Supplementary Material

Figure 1: Result of viable cell numbers that were concurrently evaluated during the cell cycle analysis after synchronization at G1/S boundary.
Figure 2: Result of viable cell numbers that were concurrently evaluated during the cell cycle analysis after synchronization at G2/M boundary.
Figure 3: Result of viable cell numbers that were concurrently evaluated during the evaluation of cell death.
Figure 4: Result of viable cell numbers that were concurrently evaluated during Western blotting analyses (p53 & Chk1).
Figure 5: Result of viable cell numbers that were concurrently evaluated during real-time PCR analyses.

## Figures and Tables

**Table 1:** Primers for real-time PCR.

Target	Regulation	Forward	Reverse
p21	p53-regulated	5′-catgtggacctgttgctgtc-3′	5′-cctcttggagaagatcagcc-3′
ATF4	ER stress responsive	5′-ggtcagtgcctcagacaaca-3′	5′-tctggcatggtttccaggtc-3′
CHOP	ER stress responsive	5′-cttcaccactcttgaccctg-3′	5′-gagctctgactggaatcagg-3′
Cdc20	p53-regulated	5′-aatgtcctggcaactggagg-3′	5′-tgagctccttgtagtgggga-3′
Skp2	p53-regulated	5′-acctccacccagatgtgact-3′	5′-gtgctgtacacgaaaagggc-3′
Puma	p53-regulated	5′-gatctcaacgcgctgtacga-3′	5′-caggcacctaattaggctcc-3′
GAPDH	house-keeping gene	5′-ccatcaccatcttccaggag-3′	5′-gagatgatgaccctcttggc-3′
