# Supplementary material for: Classification of Five Uremic Solutes according to Their Effects on Renal Tubular Cells
Source: Int J Nephrol. 2014 Nov 9;2014:512178. doi: 10.1155/2014/512178 (PMC4241681; doi:10.1155/2014/512178)
Supplement: Supplementary file 1 — Figure 1: Result of viable cell numbers that were concurrently evaluated during the cell cycle analysis after synchronization at G1/S boundary. Figure 2: Result of viable cell numbers that were concurrently evaluated during the cell cycle analysis after synchronization at G2/M boundary. Figure 3: Result of viable cell numbers that were concurrently evaluated during the evaluation of cell death. Figure 4: Result of viable cell numbers that were concurrently evaluated during Western blotting analyses (p53 & Chk1). Figure 5: Result of viable cell numbers that were concurrently evaluated during real-time PCR analyses. [file 512178.f1.pdf]

Suppl. Fig. 1

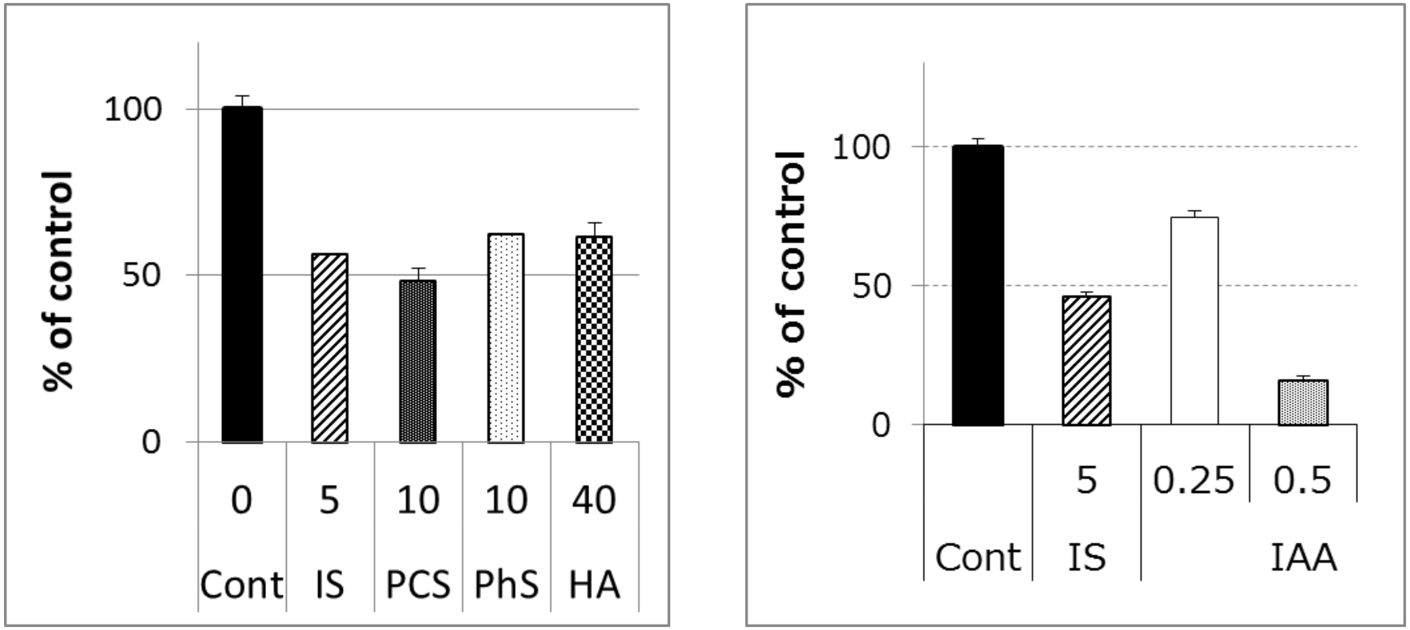

Showing the results of viable cell counts that were concurrently evaluated during the cell cycle analysis after synchronization at G1/S boundary.

Suppl. Fig. 2

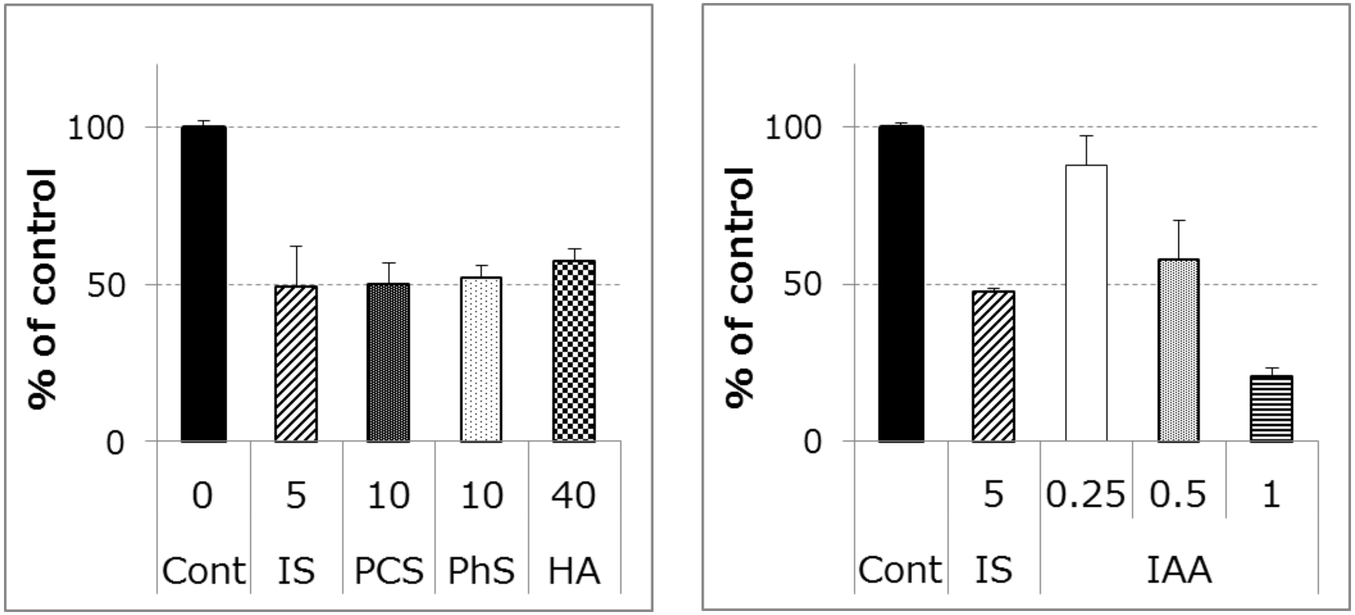

Showing the results of viable cell counts that were concurrently evaluated during the cell cycle analysis after synchronization at G2/M boundary.

Suppl. Fig. 3

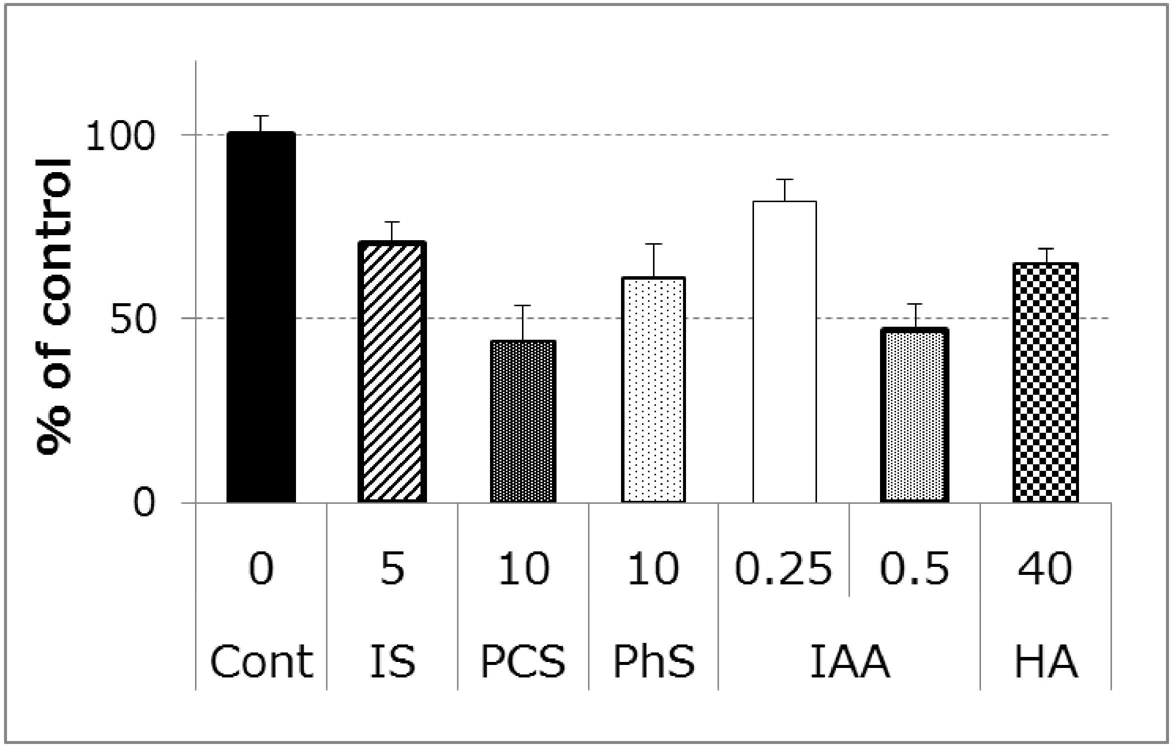

Showing the results of viable cell counts that were concurrently evaluated during the evaluation of cell death.

Suppl. Fig. 4

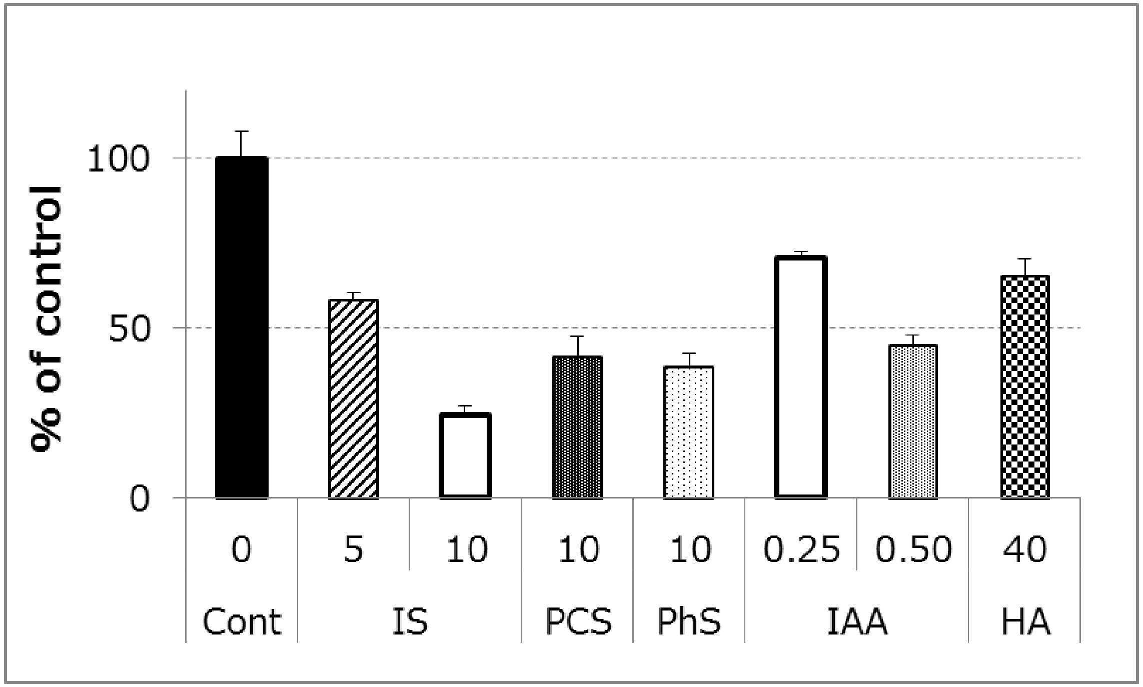

Showing the results of viable cell counts that were concurrently evaluated during Western blotting analyses (p53 & Chk1).

Suppl. Fig. 5

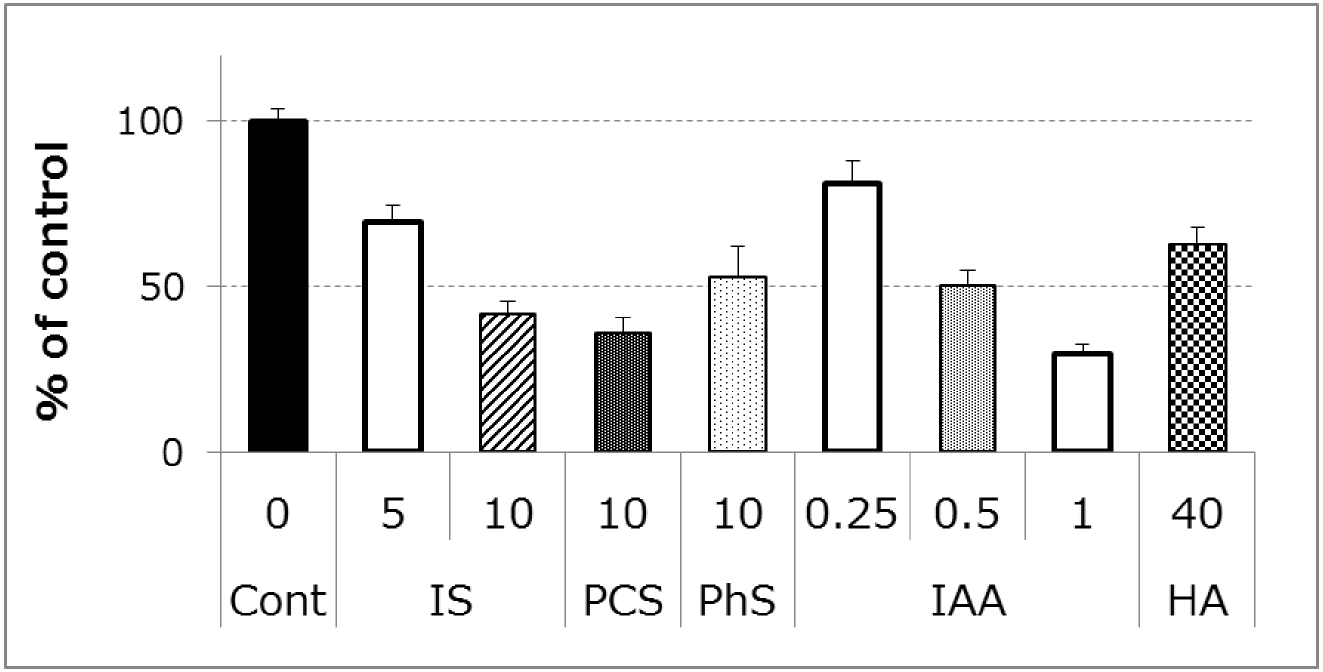

Showing the results of viable cell counts that were concurrently evaluated during real-time PCR analyses.
